# Physiology of *Pseudomonas aeruginosa *in biofilms as revealed by transcriptome analysis

**DOI:** 10.1186/1471-2180-10-294

**Published:** 2010-11-17

**Authors:** James P Folsom, Lee Richards, Betsey Pitts, Frank Roe, Garth D Ehrlich, Albert Parker, Aurélien Mazurie, Philip S Stewart

**Affiliations:** 1 Center for Biofilm Engineering and Department of Chemical and Biological Engineering, P. O. Box 173980 Montana State University - Bozeman Bozeman, Montana 59717-3980, USA; 2 Halliburton, P.O. Box 439, Pinedale, WY 82941, USA; 3 1658 Eligio Lane, Davis, CA 95618, USA; 4 Center for Genomic Sciences Allegheny Singer Research Institute 320 E. North Ave. Pittsburgh, PA 15212, USA; 5 Bioinformatics Core, Montana State University, Bozeman, Montana, USA

## Abstract

**Background:**

Transcriptome analysis was applied to characterize the physiological activities of *Pseudomonas aeruginosa *grown for three days in drip-flow biofilm reactors. Conventional applications of transcriptional profiling often compare two paired data sets that differ in a single experimentally controlled variable. In contrast this study obtained the transcriptome of a single biofilm state, ranked transcript signals to make the priorities of the population manifest, and compared ranki ngs for *a priori *identified physiological marker genes between the biofilm and published data sets.

**Results:**

Biofilms tolerated exposure to antibiotics, harbored steep oxygen concentration gradients, and exhibited stratified and heterogeneous spatial patterns of protein synthetic activity. Transcriptional profiling was performed and the signal intensity of each transcript was ranked to gain insight into the physiological state of the biofilm population. Similar rankings were obtained from data sets published in the GEO database http://www.ncbi.nlm.nih.gov/geo. By comparing the rank of genes selected as markers for particular physiological activities between the biofilm and comparator data sets, it was possible to infer qualitative features of the physiological state of the biofilm bacteria. These biofilms appeared, from their transcriptome, to be glucose nourished, iron replete, oxygen limited, and growing slowly or exhibiting stationary phase character. Genes associated with elaboration of type IV pili were strongly expressed in the biofilm. The biofilm population did not indicate oxidative stress, homoserine lactone mediated quorum sensing, or activation of efflux pumps. Using correlations with transcript ranks, the average specific growth rate of biofilm cells was estimated to be 0.08 h^-1^.

**Conclusions:**

Collectively these data underscore the oxygen-limited, slow-growing nature of the biofilm population and are consistent with antimicrobial tolerance due to low metabolic activity.

## Background

The physiological activities of bacteria growing in biofilms are difficult to divine, because these activities are diverse, change with time as the biofilm develops, and are subject to extreme micro scale spatial heterogeneity [1]. It is also clear that the metabolism and activities of a particular biofilm will be shaped by the specific chemical and physical environment in which it grows. These realities make it difficult to develop a consensus picture of the physiology of the biofilm state as there is so little overlap in the lists of genes differentially expressed between the planktonic and biofilm states of *Pseudomonas aeruginosa *prepared by different experimenters [2-7].

However, there are biofilm physiological traits, such as antimicrobial tolerance [8] and reduced growth rate [1], for which there is considerable consensus. These robust phenotypes, with their functional and evolutionary importance, should have discernable biochemical and genetic bases. We sought to understand these phenotypes with an unconventional interpretation of transcriptional profiling studies. Conventional interpretations of transcriptional profiling studies compare two paired data sets that differ in a single controlled variable (e.g., iron concentration, quorum sensing signal molecule addition). In this study, we have obtained the transcriptome for a single biofilm specimen, ranked the transcripts based on the signal intensity to make the priorities of the population manifest, and compared rankings for *a priori *identified physiological marker genes between the biofilm and a number of published data sets. For example, if we wish to discern whether the biofilm is responding to iron limitation, we first identify a set of genes that are up-regulated in response to iron deprivation (e.g. the work of Ochsner [9]). The rank of each of these transcripts in the biofilm data set is then compared to transcript ranks for the same genes in data sets collected from both rapidly growing and deliberately iron-starved cultures. In this way it becomes possible to evaluate physiological activities in the biofilm rather than just documenting differences between the biofilm and a reference state.

In the experiments reported here, RNA was extracted from an entire, homogenized biofilm specimen. An obvious concern with this approach is that it neglects the inherent biological heterogeneity of the biofilm [1]. We would like to address this concern upfront with two points. First, just because a population is heterogeneous does not mean that measurements of population averages are invalid. Population averages are very widely and informatively used in biology. Second, we suggest that even the concept of an average may not be appropriate in this case. The current conceptual model of *P. aeruginosa *drip-flow biofilms is that they consist of two distinct populations: an aerobic, metabolically active upper layer and a lower, and larger, layer consisting of inactive cells containing very low levels of mRNA [10,11]. Because the inactive cells contain so little RNA, this majority is expected to be essentially invisible on the microarray. From this perspective, the transcriptomes reported here may best be thought of as reflecting the properties of the transcriptionally-active subpopulation rather than the average behavior of the entire population. These concepts are elaborated on in the Results and Discussion.

## Results and Discussion

Three day old drip flow biofilms of *P. aeruginosa *were characterized with respect to antibiotic tolerance, oxygen availability, and microscale patterns of protein synthetic activity. These biofilms contained 9.56 ± 0.31 cfu cm^-2^.

### Reduced antibiotic susceptibility of biofilm bacteria

*P. aeruginosa *cells grown in biofilms were protected from killing by tobramycin and ciprofloxacin, in comparison to actively growing planktonic bacteria. Both antibiotics rapidly and effectively reduced viable cell numbers in an aerobic, planktonic culture. After 12 h of treatment with 10 μg ml^-1 ^tobramycin or 1.0 μg ml^-1 ^ciprofloxacin, planktonic log reductions measured were 3.18 ± 1.79 (n = 3, ± SD) and 4.84 ± 0.55 (n = 3, ± SD) for tobramycin and ciprofloxacin, respectively. In contrast, neither antibiotic was very effective against biofilms of *P. aeruginosa*. After 12 h exposure to antibiotic in continuously flowing medium, the log reductions in viable cell numbers were 0.72 ± 0.56 (n = 3, ± SD) and 1.37 ± 0.06 (n = 3, ± SD) for tobramycin and ciprofloxacin, respectively. The log reductions measured for biofilm bacteria were 23% and 28% of the planktonic log reductions for the two antibiotics, respectively. Reduced killing of the biofilm in comparison to planktonic cells was statistically significant (*p *= 0.04 and *p *= 0.0004 for tobramycin and ciprofloxacin, respectively). These data demonstrate that these drip-flow biofilms exhibit the antibiotic-tolerant phenotype that is considered a hallmark of the biofilm mode of growth.

When biofilm bacteria were dispersed prior to antibiotic exposure, they again became susceptible to the antibiotics. Log reductions measured for biofilm cells re-suspended into aerated medium and treated with tobramycin or ciprofloxacin for 12 h were 3.90 ± 0.10 and 4.40 ± 0.53, respectively. This degree of killing was the same as that measured for planktonic bacteria, indicating that susceptibility was rapidly and fully restored upon dispersal of cells from the biofilm.

### Low oxygen concentrations in biofilms

**Figure 1:**
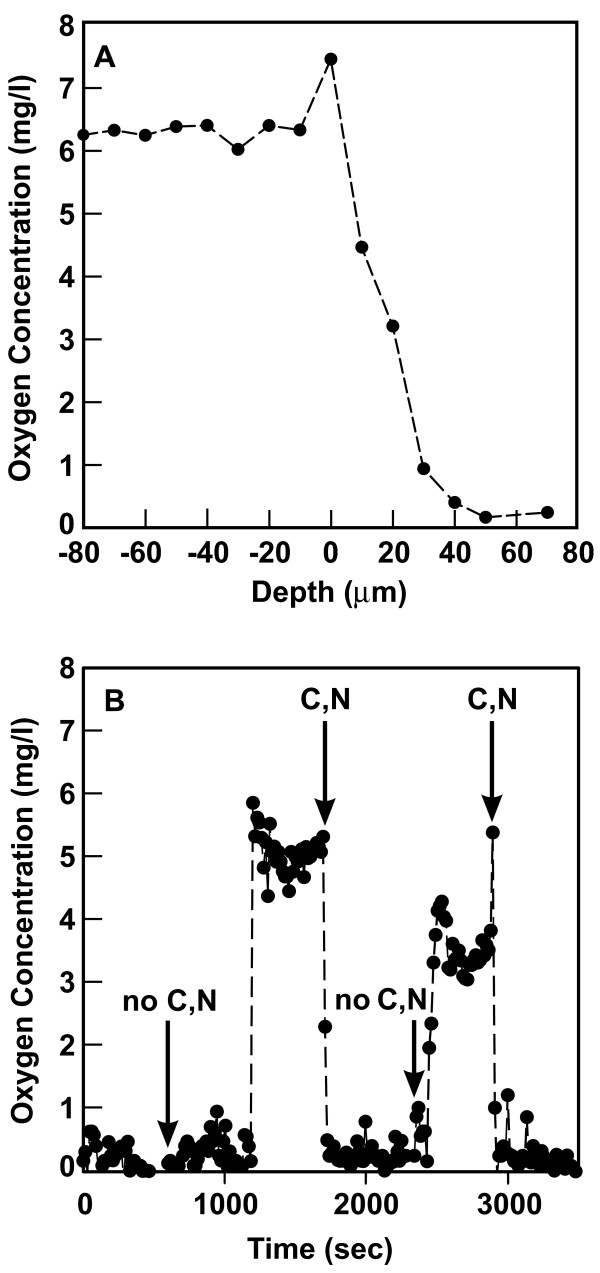
**Oxygen concentrations in *Pseudomonas aeruginosa *biofilms**. Panel A shows a representative oxygen concentration profile with depth in the biofilm. Zero on the x-axis corresponds to the biofilm-bulk fluid interface. Negative positions are located in the fluid film above the biofilm and positive positions are located inside the biomass. Panel B shows the coupling between oxygen and glucose utilization. The oxygen microelectrode was positioned at a location within the biofilm where the oxygen concentration was low. The medium flowing over the biofilm was switched between one containing glucose and ammonium ion (C, N) and a medium lacking these constituents (no C, N) as indicated by the arrows. The complete medium is present at time zero.

An oxygen microelectrode was used to demonstrate the presence of oxygen concentration gradients in this system (Figure 1A). The oxygen concentration in the flowing fluid above the biofilm was approximately 6 mg l^-1^. Oxygen concentration decreased to 0.2 mg l^-1 ^or less inside the biofilm. A similar profile was measured in a duplicate experiment. The oxygen concentrations shown in Figure 1A may not define the lower bound of oxygen concentration inside the biofilm because the electrode was positioned only partway into the biofilm, to avoid electrode breakage.

The utilization of oxygen by bacteria is coupled to their simultaneous uptake and oxidation of a carbon source. To investigate this coupling, the oxygen microelectrode was positioned at a depth part way into the biofilm where the oxygen concentration was less than 0.5 mg l^-1 ^(Figure 1B). The medium flowing over the biofilm was then changed from complete PBM to PBM lacking glucose and ammonium sulfate. Within a few minutes after switching to this starvation medium, the oxygen concentration in the biofilm abruptly rose to approximately 5 mg l^-1^. When the complete medium containing glucose and the nitrogen source was restored, the oxygen concentration quickly dropped back to its previous low level. Upon switching once again to the starvation medium, the oxygen concentration again returned to the higher level. Restoring the complete medium again caused the oxygen concentration to fall. The same behavior was observed in a duplicate experiment. These experiments show that oxygen and glucose utilization are interdependent.

### Heterogeneous patterns of protein synthetic activity in biofilms

**Figure 2:**
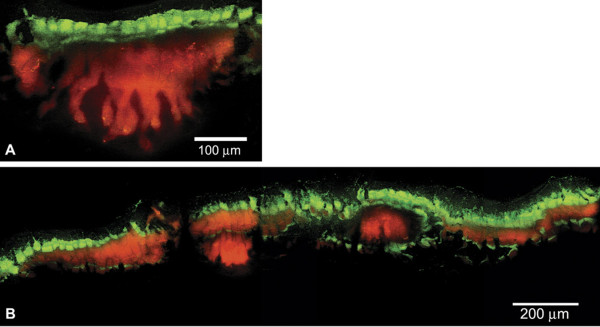
**Spatial pattern of protein synthetic activity, as revealed by transient expression of an inducible GFP (green) in a *P. aeruginosa *biofilm grown in a drip-flow reactor**. In this frozen section, the steel substratum was formerly at the bottom and the aerated nutrient medium at the top. Rhodamine B counterstaining (red) indicates the extent of the biofilm.

The induction of a GFP has been used to reveal regions of active protein synthesis in biofilms [12-14]. When this technique was applied to *P. aeruginosa *biofilms grown in drip-flow reactors, a stratified pattern of activity was observed (Figure 2). Expression of GFP was localized in a band at the top of the biofilm adjacent to the source of nutrients and oxygen. The dimension of the GFP-expressing zone averaged 66 ± 30 μm (n = 3, ± SD). The average thickness of the entire biofilm was 170 ± 78 μm (n = 3, ± SD) (Table 1). While the predominant zone of activity was along the air interface (Figure 2A), GFP fluorescence was occasionally observed in thin strata in the interior and even at the bottom of the biofilm (Figure 2B). The observation of fluorescent GFP at the bottom of the biofilm argues against the interpretation that these patterns are an artifact of incomplete IPTG penetration. In prior studies, the facile penetration of IPTG throughout *P. aeruginosa *biofilms has been demonstrated [12,14].

**Table 1:** Determination of mean biofilm thickness and mean dimension of the zone in which GFP was expressed.

Strain (plasmid)	IPTG (mM)	Biofilm*† Thickness (μm ± SD)	GFP zone*† dimension (μm ± SD)	Maximum† Fluorescence intensity (arbitrary ± SD)
PAO1 (pAB1)	0	165 ± 100	none	24 ± 26
PAO1 (pAB1)	1	170 ± 78	66 ± 30	166 ± 61
PAO1 (pMF54)	1	120 ± 38	none	3 ± 1

*The thickness of the area of GFP expression as well as the overall thickness of the biofilm was measured 3 times. Measurement of *Pseudomonas aeruginosa *PAO1 carrying plasmid pAB1 containing an IPTG-inducible GFP with and without IPTG are compared with *P. aeruginosa *carrying plasmid pMF54 lacking GFP.

†The uncertainties indicated are standard deviations.

### Transcriptional profiling of biofilms - nutritional and growth status

The RNA was extracted from 3-day old *P. aeruginosa *drip-flow reactor grown biofilms and subjected to global transcriptional profiling. These microarray data have been deposited to Gene Expression Omnibus (GEO) accession GSE22164. We compared the expression of individual genes or groups of genes indicative of specific physiological activities by analyzing the rank of the selected transcript in the drip flow biofilm transcriptome across 6 different experiments and planktonic comparator transcriptomes [15-22] listed in Table 2 and Additional file 1.

**Table 2:** *P. aeruginosa *transcriptional profiling data sets used for comparison.

GEO ID	Symbol Color	Medium	n	Reference
GSE6741	● 20% O_2 _- light green ● 2% O_2 _- gold ● 0.4% O_2 _- red ● 0% O_2 _+ nitrate - dark green	minimal amino acids 37°C, sparged and stirred exponential phase, OD ~ 0.08	2	[15]
				
GSE2430	● untreated control - pink	BHI, 37°C, shaken; early stationary phase, OD ~ 2.8	2	[18]
				
GSE4152	● untreated control - yellow ● Cu stressed - blue	MOPS buffered LB, 37°C, early exponential phase, OD ~ 0.2	2	[20]
				
GSE2885	● OD ~ 0.2 - light gray ● OD ~ 1.3 - white ● OD ~ 2.1 (Fe limited) - purple	minimal glucose, 37°C, sparged and stirred, three points in batch culture	2	[22]
				
GSE5604	● untreated control - light blue	minimal acetate, 20°C, chemostat with dilution rate 0.06 h^-1^	2	[17]
				
GSE7704	● control - brown	minimal citrate, 37°C, shaken, OD ~ 0.6	3	[19]
				
GSE5443	● control - dark blue	LB, 37°C	2	[16]
				
GSE8408	● control - dark gray	minimal succinate and non-sulfur containing amino acids, 30°C, shaken, OD ~ 0.2	3	[21]

**Figure 3:**
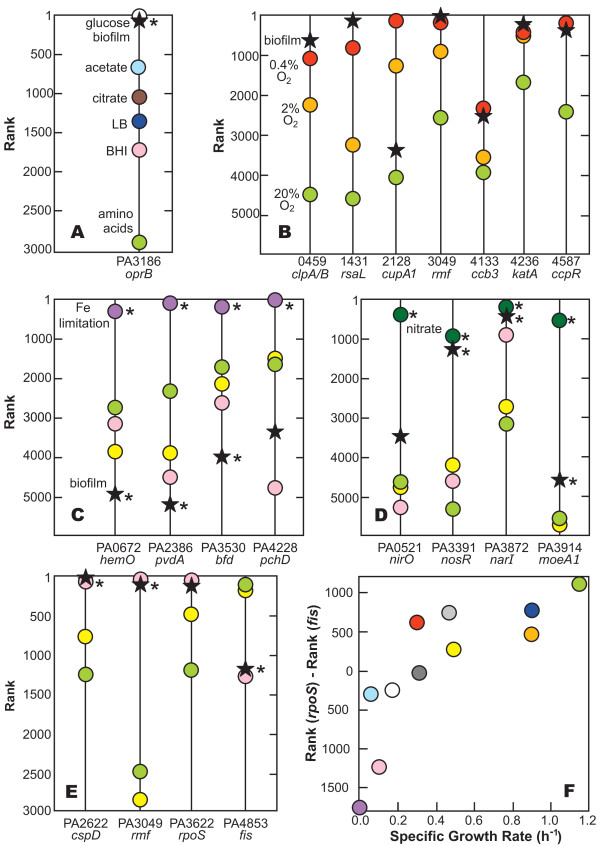
**Comparison of transcript ranks for genes related to nutritional status and growth state**. Shown are comparisons for selected genes involved in glucose uptake (A); oxygen limitation (B); iron limitation (C); presence of nitrate (D); and growth phase (E). Panel F shows the association between the difference in gene ranks for PA3622 (*rpoS*) and PA4853 (*fis*) and specific growth rate. Colored symbols correspond to individual data sets as given in Table 2 and Additional file 1. An asterisk next to a data point indicates a statistically significant difference between the indicated data set and the combined data of three standard comparator data sets (see Materials and Methods for specifics). In panel E, which concerns growth rate, the statistical comparison is to the two comparator data sets in exponential phase; the untreated control of Nalca et al was omitted. Where a label such as "Fe limitation" appears, it denotes a transcriptome that can be considered a positive control. Where no such label appears, a suitable positive control data set was lacking.

Additional file 1 contains a version of this table that includes colored symbols for visual identification of the symbols used in Figures 3, 5, and 6.

When grown on glucose, *P. aeruginosa *expresses an outer membrane protein, OprB, which is involved in the uptake of sugars [23]. Figure 3A compares the rank of the *oprB *(PA3186) transcript in several data sets, including our drip-flow reactor biofilm. This gene is highly expressed in the biofilm (n = 6, average rank of 26) and also highly expressed in one other transcriptome from a study [22] in which the bacteria were grown on a glucose-minimal medium (average of rank 7). The rank of the PA3186 transcript is lower in cells grown on minimal media supplemented with acetate or citrate, lower still on complex media such as LB or BHI, and lowest of all on a minimal amino acid medium. The straightforward interpretation of this comparison is that the strong expression of *oprB *in the drip-flow biofilm implies the presence of glucose in the system. Since the medium used in this study contained glucose as the sole carbon and energy source, these results illustrate the face validity of our approach.

**Figure 4:**
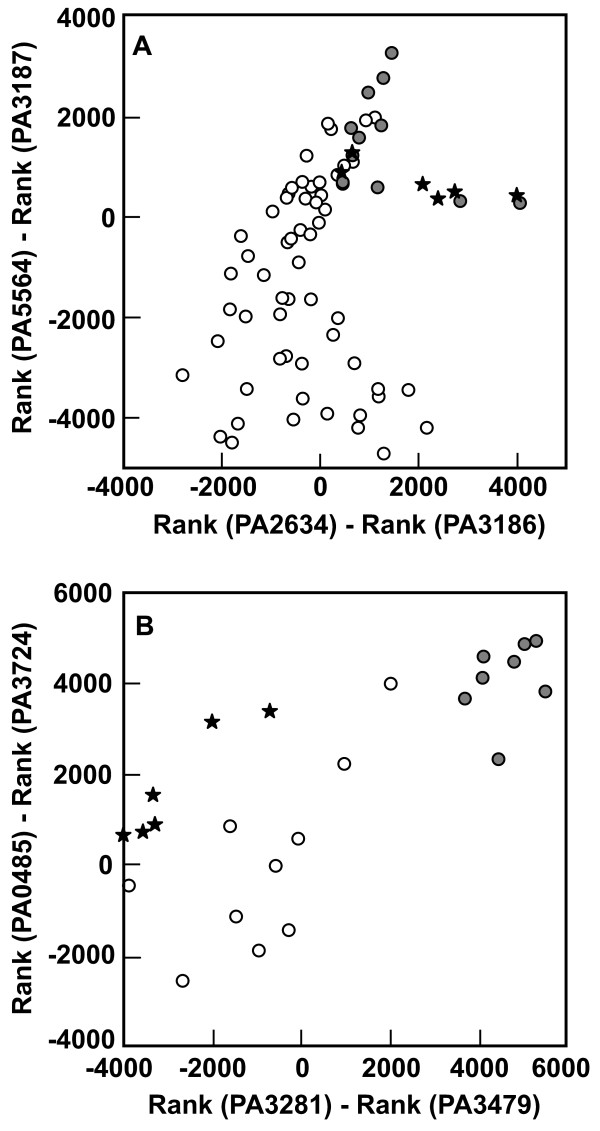
**Discrimination of glucose metabolism (A) and homoserine lactone quorum sensing (B) based on differences in transcript ranks**. Open symbols are glucose-absent or quorum sensing negative comparators in panels A and B, respectively. Filled symbols are glucose-present and quorum sensing positive comparators in panels A and B, respectively. Stars indicate drip-flow biofilm samples. The genes appearing in these graphs are annotated as: PA5564, *gidB*, glucose inhibited division protein B; PA3187, probable ATP-binding component of ABC transporter; PA2634, *aceA*, isocitrate lyase; PA3186, glucose/carbohydrate outer membrane porin OprB precursor; PA0485, conserved hypothetical protein; PA3724, *lasB*, elastase; PA3281, hypothetical protein; *rhlA*, rhamnosyltransferase chain A.

To further demonstrate the potential to diagnose metabolic activities from transcript ranks, we conducted a more comprehensive analysis of relationship between the presence or absence of glucose and the ranks of selected gene transcripts. Fifty eight samples were identified in which no glucose was present in the medium. Eleven samples were identified in which glucose was the sole or predominant carbon source. Differences in the ranks of pairs of genes, identified by inspection, were found to discriminate the glucose-present and glucose-absent data sets (Figure 4A). The drip-flow biofilm data group with the glucose-present comparators, as expected. The six glucose-absent points that overlap with the glucose-present cluster are from a single investigation in which glycerol was the predominant carbon source. The extensive commonality of pathways for catabolism of glucose and glycerol may explain this overlap.

Alvarez-Ortega and Harwood [15] identified genes induced under conditions of low oxygen concentration. From their results, we identified a subset of seven genes that were particularly strongly induced by low oxygen and whose transcript rank increased monotonically with decreasing oxygen concentration. Figure 3B compares the rank for these seven genes between drip-flow biofilms in this study and the Alvarez-Ortega and Harwood [15] data. The rankings of the transcripts for the biofilm were consistent with low oxygen concentrations for six of seven transcripts. This comparison indicates that the biofilm experienced oxygen limitation.

A recent investigation reported 117 genes induced by transferring *P. aeruginosa *from aerobic to anaerobic conditions [24]. Thirty-five genes appearing on this list also appear in Table 3, a significant overlap (*p *= 3 × 10^-12^; random chance would predict an overlap of approximately 2 genes). This overlap reinforces the interpretation of an oxygen-limited physiology of the drip-flow biofilm population.

**Table 3:** Genes expressed more highly in untreated *P. aeruginosa *drip-flow reactor biofilms (n = 6) than in several comparator transcriptomes.

		DFB*	**Comp**.*		
Gene ID	Name	Rank	Rank	Note	Description
PA0038		185	1734		hypothetical protein
PA0105-0107	*coxBA*	174	3225	O_2_	cytochrome c oxidase
PA0200		145	3494		hypothetical protein
PA0284		77	712		hypothetical protein
PA0409	*pilH*	56	539		Type IV pili biogenesis
PA0515-0519	*nirFCMS*	207	2547	O_2_	nitrite reduction
PA0523-0524	*norCB*	177	3770	O_2_	nitric oxide reductase
PA0586-0588		123	1848	O_2_	conserved hypothetical and serine protein kinase
PA0713		103	3107	O_2_	conserved hypothetical protein
PA717-0726		185	3323		hypothetical proteins of bacteriophage Pf1
PA0763	*mucA*	30	296		anti-sigma factor
PA1041		47	1850		probable outer membrane protein precursor
PA1174	*napA*	270	2433	O_2_	periplasmic nitrate reductase
PA1178	*oprH*	5	831		outer membrane protein H1 precursor
PA1190		105	3558		conserved hypothetical protein
PA1414		14	756		hypothetical protein
PA1431	*rsaL*	45	1835	O_2_	regulatory protein
PA1555-1556		64	1312	O_2_	cytochrome c oxidase
PA1592		4	67		hypothetical protein
PA1673		152	2346	O_2_	hypothetical protein
PA1746		173	2016	O_2_	hypothetical protein
PA1982-1983	*exaAB*	315	5317		alcohol dehydrogenase, cytochrome c550
PA2274		378	3473		putative monooxygenase
PA2381		64	1279	O_2_	conserved hypothetical protein
PA2485		161	2704		hypothetical protein
PA2501		133	1858		hypothetical protein
PA2622	*cspD*	19	591		stationary phase replication inhibitor
PA2754		140	1797		conserved hypothetical protein
PA2780-2781		205	2983		hypothetical proteins
PA2807-2808	*ptrA*	47	3378	Cu	hypothetical and two-component repressor
PA2862	*lipA*	176	2785		lactonizing lipase precursor
PA2883		121	2207		hypothetical protein
PA2937		53	3144		hypothetical protein
PA3040		40	553		conserved hypothetical protein
PA3049	*rmf*	11	1575	O_2_	ribosome modulation factor
PA3126	*ibpA*	134	1382		heat-shock protein IbpA
PA3181		226	2020		2-keto-3-deoxy-6-phosphogluconate aldolase
PA3186-3190	*oprB*	68	1936		glucose uptake
PA3205		16	1033		hypothetical protein
PA3235		101	2118		conserved hypothetical protein
PA3309		33	1076	O_2_	universal stress protein
PA3369-3371		196	3302		hypothetical proteins
PA3412		163	3772	Cu	probable transcriptional regulator
PA3415		225	2682		probable dihydrolipoamide acetyltransferase
PA3416-3417		259	2723		pyruvate dehydrogenase
PA3418	*ldh*	52	2352		leucine dehydrogenase
PA3515-3519		92	2804	Cu	hypothetical proteins and probable lyases
PA3523		128	3765	Cu	probable RND efflux membrane fusion protein
PA3552	*pmrH*	269	2400		lipopolysaccharide modification
PA3572		107	1616		hypothetical protein
PA3577		253	3216		hypothetical protein
PA3690-3691		57	1482	Cu	probable metal-transporting P-type ATPase
PA3792	*leuA*	38	603		2-isopropylmalate synthase
PA3819		44	508		conserved hypothetical protein
PA3920		79	2577	Cu	probable metal transporting P-type ATPase
PA3973		292	2829		probable transcriptional regulator
PA4352		114	1316	O_2_	conserved hypothetical protein
PA4550-4551	*fimUpilV*	167	2088		type 4 fimbrial biogenesis proteins
PA4577		124	2292		hypothetical protein
PA4607-4611		136	2173		hypothetical proteins
PA4739		143	1345	O_2_	conserved hypothetical protein
PA4773,4776	*pmrB*	166	2538		hypothetical and two-component sensor
PA4781-82		202	2538		probable two-component response regulator
PA5100	*hutU*	258	2660	O_2_	urocanase
PA5212		29	881		hypothetical protein
PA5427	*adhA*	112	2664	O_2_	alcohol dehydrogenase
PA5446		7	866		hypothetical protein
PA5460		118	3962		hypothetical protein
PA5475		294	2787	O_2_	hypothetical protein

*Average Rank in drip flow biofilms (DFB) and three comparator microarray data sets (Comp. Rank) named in the Materials and Methods.

We identified four genes strongly up-regulated by iron limitation [9] and compared their expression between drip-flow biofilm, three standard comparison data sets [15,18,20], and a positive control in which the bacterial culture was deliberately iron-limited (Figure 3C) [22]. All four genes were highly ranked in the iron-limited positive control. The expression rank of these four genes in the drip flow biofilm was consistently lower in comparison to the reference data sets. These data suggest that bacteria in the drip-flow biofilm as grown in this study did not experience limitation for iron. The concentration of iron in the medium, added in the form of ferrous ammonium sulfate, was 1.5 μM.

From the literature, we identified four genes that are induced by the presence of nitrate in the medium, either under aerobic or anaerobic conditions [25]. The expression rank of these genes is compared in Figure 3D. The rank for the drip-flow biofilm for all four genes was higher than the three standard comparison data sets and lower than a nitrate-amended positive control. The medium used to grow the biofilm did not contain added nitrate.

Figure 3E presents a comparison of gene rank for four growth phase responsive genes. Three genes associated with stationary phase, *cspD*, *rmf*, and *rpoS*, [26-29] were very highly ranked in both our drip flow biofilm and the comparison data set that was sampled in stationary phase. The fourth gene whose expression is associated with early exponential phase growth, *fis*, [26,29] showed the inverse ranking. The biofilm and stationary phase culture had similar ranks for the *fis *gene, while the two systems in which bacteria were rapidly growing had much higher ranks. These comparisons suggest that many of the cells in the biofilm exhibit stationary phase character.

To further explore the potential relationship between transcript levels for these genes and growth state, we plotted gene rank for *fis *and *rpoS *as a function of specific growth rate, where a growth rate was reported or optical density versus time data permitted a quantitative estimation (not shown). Though the data is somewhat noisy, it is clear that *rpoS *gene rank decreases with increasing growth rate. The rank of the *fis *gene is relatively constant above a specific growth rate of approximately 0.2 h^-1^, and decreases below this growth rate. The difference in gene rank between *rpoS *and *fis *increases with specific growth rate (Figure 3F). This analysis points to the possibility of inferring growth rate from transcriptomic data. For example, in the drip-flow biofilm the difference in *rpoS *and *fis *gene rank was -1135 ± 296 (n = 6, ± SD). From Figure 3F, this difference corresponds to a specific growth rate of approximately 0.08 h^-1^. Taking the results of Figures 3E and 3F together, it appears as if bacteria in the biofilm were growing very slowly.

### Oxygen availability limits growth in biofilm

In this experimental system, two potentially limiting substrates for bacterial growth were glucose and oxygen. The composition of the medium used ensured excess nitrogen, phosphorous, sulfur, and other elemental requirements. For example, the molar ratio of ammonium to glucose carbon was 2.3, which provided approximately ten-fold excess nitrogen.

There is no basis for anticipating that glucose was limiting in any part of the biofilms that were grown in this study. This can best be appreciated by a simple calculation. As derived by Williamson and McCarty [30], the metabolic substrate that will first be depleted in a biofilm can be determined by calculating the dimensionless quantity *D*_eG_*S*_G_/*D*_eO2_*S*_O2_*Y*_GO2. _This ratio is a measure of the relative diffusive fluxes of glucose and oxygen into the biofilm, where *D*_e _denotes the effective diffusion coefficient of the respective substrate in the biofilm, *S *denotes the bulk fluid concentration of the respective substrate, and *Y*_GO2 _is the stoichiometric coefficient relating the consumption of glucose and oxygen. In the present case, we take the effective diffusion coefficients of oxygen and glucose to be 1.53 × 10^-5 ^cm^2 ^s^-1 ^and 2.69 × 10^-6 ^cm^2 ^s^-1^, respectively [31]. The yield coefficient has been carefully measured, in biofilms of this bacterium, and is 2.25 g glucose per g oxygen [32]. With the bulk fluid concentration of glucose at 200 mg l^-1 ^and the bulk fluid concentration of oxygen at 6 mg l^-1^, the quantity given by the ratio above has a value of 2.6. This value being greater than 1 means that glucose is provided in excess and that oxygen is the limiting substrate. This interpretation is consistent with the strong expression of *oprB *in biofilm specimens (Figure 3A) and the analysis shown in Figure 4A.

Microelectrode measurements provided direct chemical evidence of reduced oxygen availability (Figure 1). Steep oxygen concentration gradients were measured in the vicinity of the biofilm, with parts of the biofilm experiencing oxygen concentrations of 0.2 mg l^-1 ^or less (Figure 1). These measurements are concordant with the transcriptomic analysis of biofilm bacteria that provides direct biological evidence of oxygen limitation (Figure 3B, Table 3). The following describes our physical understanding of the concentration gradients in this particular biofilm system. In the aerobic layer, both oxygen and glucose are consumed. Once the oxygen has been depleted, utilization of glucose stops. Abundant glucose, approximately 125 mg l^-1^, is predicted to be available at the bottom of the biofilms studied in this investigation.

We note that *P. aeruginosa *is unable to ferment glucose and no arginine was present, precluding fermentative growth [33,34]. No alternative electron acceptor, such as nitrate, was added to the medium used in these studies. Therefore, growth by denitrification was also precluded. The expression of genes associated with denitrification in the biofilm (Figure 3D, Table 3) may have been a response to oxygen limitation. In summary, once oxygen was depleted in this system, one would predict that growth would cease.

### Biofilm harbors slowly-growing or non-growing bacteria

We hypothesize that oxygen limitation in *P. aeruginosa *drip-flow biofilms resulted in slow growth or lack of growth of many of the bacteria in the biofilm. The expression of an inducible GFP was focused in a sharply demarcated band immediately adjacent to the oxygen source. This band represented approximately 38% of the biofilm, indicating that as much as 62% of the biofilm could be anoxic and anabolically inactive. Because alternative fermentable substrates or electron acceptors were absent, oxygen limitation is expected to be sufficient to lead to arrested growth in anoxic regions of the biofilm. This interpretation is qualitatively consistent with previous studies of oxygen availability and spatial patterns of physiological activity in some other *P. aeruginosa *biofilms [12-14,35,36].

Transcriptomic data show that the biofilm exhibited stationary phase character (Figure 3E). This is evident in the pronounced expression of *rmf*, a stationary-phase inhibitor of ribosome function [37], *cspD*, a stationary-phase inhibitor of replication [38], and *rpoS*, a stationary-phase sigma factor[27]. In a previous investigation, we independently reported the elevated expression of *rpoS *in *P. aeruginosa *biofilms [39]. A gene associated with early exponential phase growth, *fis*, was expressed at relatively low levels, consistent with very slow growth. Our estimate of an average specific growth rate of 0.08 h^-1 ^is approximately ten percent of the specific growth rate of *P. aeruginosa *in this medium of 0.74 h^-1^. Colony biofilms of a mucoid strain of *P. aeruginosa *had a reported specific growth rate that was two percent of the maximum specific growth rate in that system [13].

Here we consider two alternative conceptual models for growth and activity within the biofilm. These models attempt to address the microscale heterogeneity that is obviously present and which the transcriptional analysis is incapable of resolving. Both of these conceptual models view the biofilm as having two layers of differing growth rates. In the first model, an aerobic layer representing the upper 40% of the biofilm grows at 0.2 h^-1 ^while the bottom layer has a specific growth rate of zero. The population average growth rate (0.4*0.2 h^-1 ^+ 0.6*0 h^-1^) would be 0.08 h^-1^. In the second model, an aerobic layer representing the upper 40% of the biofilm grows at 0.08 h^-1 ^while the bottom layer has a specific growth rate of zero. The population average growth rate would be 0.032 h^-1^. We believe that the second model is the more realistic. The transcriptome obtained in this study does not represent the average behavior of the biofilm. It reflects rather the activities of the transcriptionally-active subpopulation, which is the aerobic upper layer. Localized gene expression measurements performed by microdissection and PCR show that the *rpoS *transcript is more abundant in the upper layer of the biofilm compared to the middle or bottom layers [10,11]. This confirms that the "active" cells in the biofilm are in fact in a stationary phase-like state and that the inactive cells are depleted of most mRNA.

### Transcriptional profiling of biofilms - stress responses and quorum sensing

The same approach of comparing ranks of selected genes indicative of specific physiological activities was applied to examine oxidative stress, copper stress, efflux pump activities, and quorum sensing in drip-flow biofilms.

**Figure 5:**
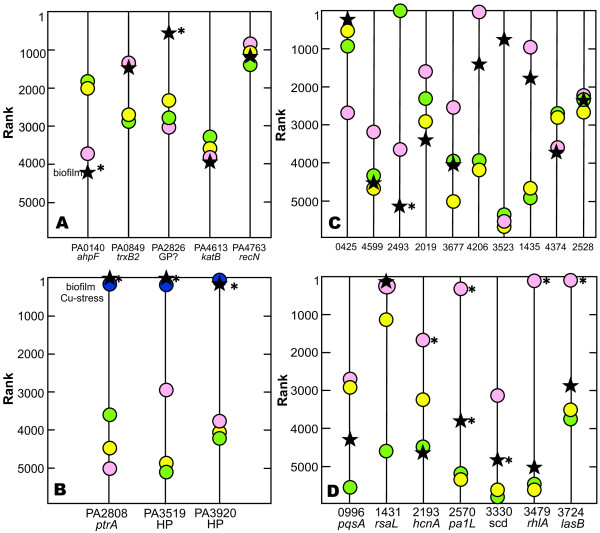
**Comparison of transcript ranks for genes involved in stress responses and quorum sensing**. Shown are comparisons for selected genes involved in oxidative stress (A); copper stress (B); efflux pumps (C); and homoserine lactone quorum sensing (D). Symbols correspond to individual data sets as given in Table 2 and Additional file 1. An asterisk next to a data point indicates a statistically significant difference between the indicated data set and the combined data of three standard comparator data sets (see Materials and Methods for specifics). Where a label such as "Cu stress" appears, it denotes a transcriptome that can be considered a positive control. Where no such label appears, a suitable positive control data set was lacking.

The expression levels, as quantified by transcript rank, of five genes associated with oxidative stress [40-42] were not in general elevated in reference to the comparators (Figure 5A). The only possible exception, a putative glutathione peroxidase (PA2826), is difficult to interpret clearly since this gene is also induced under copper stress (see the next paragraph). Thus we conclude that no unusual oxidative stress is occurring.

We noticed that several genes associated with copper stress, as reported by Teitzel et al. [20], were highly expressed in drip-flow biofilms (Figure 5B). The nominal copper concentration in PBM is 0.16 μM, which is much less than the 10 mM Teitzel et al. used. We identified another data set, that of Love and co-workers [17], in which an acetate minimal medium was supplemented with trace elements including Cu at a final concentration of 2.9 μM. Copper stress genes were highly expressed in this case as well. We therefore suggest that micro molar concentrations of copper are sufficient to induce a copper stress response when *P. aeruginosa *is grown in minimal media.

Efflux pumps were not up-regulated in *P. aeruginosa *biofilms in general (Figure 5C). The one instance of obvious high level expression, PA3523, is associated with copper stress [20].

Three different laboratories have published data on the set of genes regulated by homoserine lactone quorum sensing in *P. aeruginosa *[43-45]. We selected a consensus subset of seven of these genes that are more highly expressed under conditions of active quorum sensing and compared the drip-flow biofilm transcriptome to the standard reference data sets (Figure 5D). The biofilm rank was relatively low for all but one of these genes, PA1431 or *rsaL*. Though *rsaL *is itself quorum sensing activated, the *rsaL *gene product is a negative regulator that represses many other quorum-sensing activated genes [46]. Thus the high level expression of *rsaL *may be consistent with repression of many of the other genes shown in Figure 5D. These data show, surprisingly, that homoserine lactone quorum sensing is not active in these drip-flow biofilms.

To further demonstrate the potential for differences in transcript ranks to serve as indices of specific physiological activities, homoserine lactone quorum sensing was examined in a fashion analogous to that described above for glucose (Figure 4A) and growth rate (Figure 3F). The eight quorum sensing positive samples plotted in Figure 4B are planktonic cultures with optical densities greater than 2.0. The 10 quorum sensing negative samples in this figure are either from quorum sensing deficient mutants or planktonic cultures of very low optical density. The drip-flow biofilm data points clearly do not group with quorum sensing positive benchmarks (Figure 4B).

Quorum sensing has been associated with biofilm development in *P. aeruginosa *by many investigators [47-50], so our finding that this communication system is silent in three-day old drip-flow biofilms seems at odds with the literature. This result is internally consistent, however, with the elevated expression of two negative regulators of quorum sensing, *rsaL *[46] and *algR*, another repressor of quorum sensing [51]. The *algR *gene transcript ranked 252 in drip-flow biofilms and 1251 in the same comparator data sets used to compile Table 3. We speculate that quorum sensing may have been active at an earlier stage of biofilm formation in the drip-flow reactor.

### Transcriptional profiling - biofilm extracellular matrix genes

**Figure 6:**
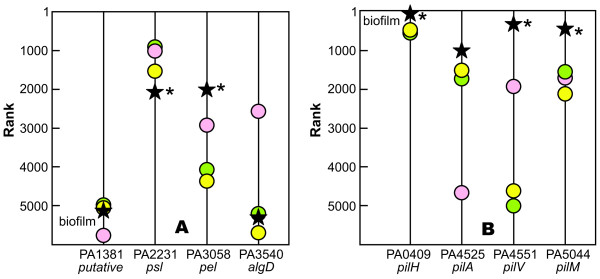
**Comparison of transcript ranks for selected genes involved in synthesis of extracellular polysaccharides (A) and production of pili (B)**. Symbols correspond to individual data sets as given in Table 1. An asterisk next to a data point indicates a statistically significant difference between the indicated data set and the combined data of three standard comparator data sets (see Materials and Methods for specifics).

Extracellular polysaccharides and proteins are common constituents of the biofilm matrix. There are four putative or known polysaccharide biosynthetic operons in *P. aeruginosa *[52]. Both *pel *and *psl *genes were expressed in the biofilm while alginate biosynthetic genes were not. Only the *pel *genes were up-regulated in biofilms compared to the three planktonic controls (Figure 6A). The low level of expression of *algD *in the drip-flow biofilm is consistent with prior reports that alginate is not an important constituent of PAO1 biofilms [53]. Our transcriptomic data suggest that the pel and psl polysaccharides may be important constituents of the extracellular matrix of drip-flow biofilms while alginate is unimportant (Figure 6A). The rank of the *cdrA *gene, a recently described adhesin that interacts with the *psl *polysaccharide [54], was not much different in drip-flow biofilms and planktonic comparators.

Genes associated with the elaboration of type IV pili were strongly expressed in drip-flow biofilms (Figure 6B). This has led us to speculate that these extracellular proteinaceous appendages contribute to the mechanical stability of the biofilm rather than motility, perhaps by binding to extracellular DNA [55,56].

### Transcriptional profiling - independent identification of upregulated genes in biofilms

All of the preceding analyses were predicated using *a priori *identification of a set of genes associated with discrete physiological conditions. The comparison of transcript ranks can also be used to identify genes that are differentially regulated between the drip-flow biofilm data set and planktonic comparator data sets. Table 3 reports the 100 genes that ranked more highly in the drip-flow biofilm than in the comparator data set, by fold-changes in rank ranging from 8 to more than 100. Some of the salient features of this list are genes associated with oxygen limitation (27 genes), copper stress (12 genes), bacteriophage Pf1 (10 genes), denitrification (8 genes), ethanol metabolism (4 genes), and three genes involved in type IV fimbrial biogenesis. Seven of the genes listed in Table 3 (PA0200, PA0409, PA0713, PA1174, PA3309, PA3572, PA5446) appear on the consensus list of gene transcripts upregulated in *P. aeruginosa *biofilms reported by Patell et al [7].

### Biological basis of biofilm antibiotic tolerance

*P. aeruginosa *strain PAO1 formed biofilms in the drip-flow reactor that were poorly killed by tobramycin or ciprofloxacin. This result is concordant with many previous investigations of antibiotic susceptibility of *P. aeruginosa *biofilms developed in other in vitro systems [12,13,43,57-82].

A plausible and long-standing explanation for reduced antibiotic susceptibility in biofilms is that nutrient limitation leads to slow growth or stationary phase existence for many of the cells in a biofilm, reducing their antimicrobial susceptibility [63,83-85]. This mechanism is consistent with all of our data. Multiple lines of evidence support oxygen limitation and arrested growth in drip-flow biofilms: oxygen concentration gradients (Figure 1), expression of genes associated with oxygen limitation (Figure 3B, Table 3) and stationary phase existence (Figure 3E), and stratified patterns of protein synthetic activity (Figure 2). In a previous study using a different in vitro biofilm model, we reported that oxygen limitation could account for 70 percent or more of the protection from six antibiotics observed in *P. aeruginosa *colony biofilms [12]. A recent report showed that ciprofloxacin and tetracycline preferentially killed the metabolically active subpopulation in *P. aeruginosa *biofilms [65].

Oxygen limitation is known to occur in vivo in cystic fibrosis patients [86]. Further, molecular biological evidence suggests that *P. aeruginosa *in the cystic fibrosis lung experiences anaerobic conditions [87]. In an investigation of in situ growth rates of *P. aeruginosa *obtained from chronic lung infections, approximately 11% of cells were determined to be in a non-growing stationary-phase based on their ribosome content [88]. The average specific growth rate of the growing bacterial cells was 0.31 h^-1^. This shows that a non-growing population may be relevant in vivo, though it suggests that the population of bacteria in the infected lung were overall more active than we describe here for drip-flow biofilms.

### Heterogeneity within the biofilm

Here we remark on the "averaging" that occurs when the entire biofilm is mashed up and extracted RNA is analyzed. This method mixes together the RNA from transcriptionally active cells in the aerobic upper layer of the biofilm with RNA from inactive bacteria in the lower layers of the biofilm. The result is not a simple average of the activities of the two layers because there is so much less mRNA in the inactive bacteria. Indeed, the inactive bacteria may contribute little to the overall microarray signal. For this reason, the transcriptome that has been examined in this work may best be thought of as representing the transcriptionally-active supopulation of bacteria rather than an average of the entire biofilm population.

A recently described laser capture microdissection technique provides a direct experimental approach for quantifying the amount of specific RNA sequences in distinct regions of the biofilm [10,11]. This method begins with cryoembedding an intact biofilm and preparing frozen cross sections. Small user-defined areas of the cross section can be physically removed and amplified by PCR to detect specific transcripts. Application of this approach to drip-flow *P. aeruginosa *biofilms has revealed that the upper layer of the biofilm is enriched in mRNA compared to the lower layers [10,11]. For example, in drip-flow biofilms the number of RNA copies of the housekeeping gene *acpP *was approximately 60 times smaller at the bottom of the biofilm compared to the top [10]. For the *rhlR *transcript the difference between top and bottom was approximately 30-fold [11].

### Utility of ranked transcriptome analysis

Conventional transcriptional profiling is applied to paired samples and allows for the discovery of genes that are differentially regulated between the two samples. For example, comparing the transcriptomes of samples grown at two different temperatures or in the presence and absence of a signaling molecule leads directly to the identification of genes regulated by temperature or by the specific signal chemistry. This is the usual usage of transcriptional profiling technology.

In this investigation, we sought to use transcriptional profiling to provide insight about the physiological activities of a single sample. Rather than chronicling the differences between two conditions (e.g., biofilm and planktonic), we wanted to ask and answer the question "What is the transcriptionally active biofilm cell doing?" To do this, we ranked the transcriptome, which makes manifest the priorities of the cell, at least at the transcriptional level. To interpret this ladder of genes, we independently identified from the literature sets of genes as markers of particular physiological activities and then compared the ranks of these genes to the ranks in several planktonic comparator transcriptomes. As the public database of transcriptional data expands, this approach becomes more and more feasible and powerful. Our effort is a preliminary one that surely will benefit from many improvements.

## Conclusions

The physiological activities of mature *P. aeruginosa *biofilms were elucidated by integrating existing knowledge of gene functions and transcriptional responses, a public database of transcriptomic data, a whole-biofilm transcriptome, and other chemical and biological assay results. The biofilm was found to be limited for oxygen, growing slowly, and exhibiting stationary phase character.

## Methods

### Bacterial strains and growth conditions

Pure cultures of the *Pseudomonas aeruginosa *strain PAO1 were used for all experiments involving antibiotic treatment. Experiments investigating patterns of protein synthetic activity, used strain PAO1 (pAB1), containing a plasmid with an IPTG inducible gene for expression of a stable GFP. The vector control *P. aeruginosa *PAO1 (pPMF54) contained the same plasmid as pAB1 without the GFP gene. *P. aeruginosa *was grown in *Pseudomonas *basal mineral medium [89] (PBM) containing 0.2 g l^-1 ^glucose for experiments measuring growth or antibiotic susceptibility. Inocula were grown in the same medium containing 1 g l^-1 ^glucose. Cultures were prepared in shake flasks at 37°C with 200 rpm agitation. Tobramycin sulfate was obtained from Sigma-Aldrich, ciprofloxacin hydrochloride was a gift of the Bayer Corporation. Viable cell numbers were determined by colony formation on tryptic soy agar (TSA; Becton Dickinson).

### Preparation of biofilms

Biofilms were grown in drip-flow reactors as described [36] using PBM supplemented with 0.2 g l^-1 ^glucose. Drip-flow reactors consisted of four parallel chambers that were covered with polycarbonate windows containing septa for the introduction of media using 22 gauge needles, and a filtered air vent. Media was pumped into the chambers at a flow rate of 60 ml h^-1^, dripping onto the stainless steel slides (8.5 cm × 1.3 cm) placed in the chambers. The reactors were placed on a stand inclined at 10° from horizontal and PBM would flow the length of the coupon and drain from the reactor. The reactors were inoculated by adding 1 ml of an overnight culture to 15 ml of fresh PBM used to cover the slides (inoculum OD_600 _≈ 0.3) in PBM (1 g l^-1 ^glucose). The reactor was sealed by clamping the effluent tubes and the inoculum was allowed to sit in the reactor for 18-24 h on a level surface. After the inoculation period, the reactor was inclined and flow was initiated. The entire drip-flow reactor was kept in a 37°C incubator. Medium flowing from outside the incubator was warmed by passing the silicone tubing through a grooved aluminum block kept in the incubator. The biofilms were grown in the drip flow reactors for 72 hours after the static inoculation phase.

### Biofilm protein synthetic activity patterns

*P. aeruginosa *PAO1 (pAB1) biofilms were grown for 72 hours in drip flow reactors. The medium was then supplemented with 1 mM IPTG and flow continued for 4 h. After this induction period, biofilm-covered slides were removed from the reactor and cryo-embedded in Tissue-Tek O.C.T. (VWR Scientific). Cryo-embedded biofilms were cryo-sectioned, and examined by confocal laser scanning microscopy with a Leica TCS NT with excitation at 488 nm and emission filter of 500 - 530 nm. Dimensions of the biofilm and the GFP-expressing zone were determined by image analysis using Scion Image software (Scion). Some specimens were counterstained with rhodamine B following IPTG induction of the GFP. In these cases, rhodamine B was introduced into the medium at a concentration of 5 μg ml^-1 ^for 30 min. The biofilms were then rinsed with fresh medium for 30 min before cryo-embedding.

### Oxygen concentrations in biofilms

Oxygen concentration profiles in biofilms were measured with microelectrode technology described in detail elsewhere [90,91]. The microelectrode manipulator was placed inside the incubator so that the measurements could be made at 37°C.

### Antibiotic susceptibility of biofilms

After 72 hours of growth in the absence of antibiotic, the desired antibiotic was added to the growth medium, and the flow continued for an additional 12 hours. Tobramycin was applied at 10 μg ml^-1 ^and ciprofloxacin at 1.0 μg ml^-1^. After treatment the stainless steel coupons were removed from the reactor and the number of viable cells was determined by scraping the biofilms into 9 ml of phosphate buffer (pH 7.2, 1.4 mM) and homogenizing for 1 min. The resulting cell suspensions were serially diluted and plated on TSA. Killing was reported as a log reduction. The log reduction was calculated relative to the cell count at time zero. Experiments were performed at least in triplicate.

### Re-suspended biofilm and planktonic susceptibility

The antibiotic susceptibility of log phase (OD_600 _0.030 - 0.08) and re-suspended biofilms of *P. aeruginosa *was determined. One milliliter of an overnight culture of *P. aeruginosa *PAO1 was sub-cultured into 29 ml of PBM (1 g l^-1 ^glucose) and grown overnight with agitation (37°C, 200 rpm) prior to exposure to antibiotics. One milliliter aliquots from the overnight cultures were mixed with 29 ml of fresh PBM (1 g l^-1 ^glucose) containing antibiotics (tobramycin at 10 μg ml^-1 ^or ciprofloxacin at 1.0 μg ml^-1^) to start treatment. Biofilms (72 h) scraped from coupons, were homogenized in phosphate buffer for 1 minute using a tissue homogenizer and re-suspended in 30 ml of PBM (1 g l^-1 ^glucose) with antibiotics (as above), to yield a cell density of 3.0 × 10^7 ^cells ml^-1^. After suspension in antibiotic containing media, cultures were placed in an orbital shaking incubator at 37°C and sampled over the course of 12 hours. The resulting cell suspensions were serially diluted and viable bacterial numbers were determined by plating on TSA.

### Preparation of biofilms for RNA extraction

Biofilms were grown in the drip flow reactor for either 72 h (n = 3) or 84 h (n = 3). Data from these two time points were pooled. Biofilms were scraped directly into 1 ml of RNA*later*^® ^(Ambion). Clumps were dispersed by repeated pippetting with a micro-pipette and the recovered biofilms were stored at 4°C for one day prior to removal of the RNA*later*^® ^by centrifugation (15 min, 4°C, and 14000 g) and freezing of the biofilm cells at -70°C.

### RNA extraction

Biofilm cells were thawed on ice and re-suspended in 300 μl of 1 mg lysozyme ml^-1 ^Tris-EDTA buffer (TE; 10 mM Tris, 1 mM EDTA, pH 8.0) and divided into three aliquots. Each aliquot was sonicated for 15 s, and incubated at room temperature for 15 minutes. RNA was extracted with an RNeasy^® ^mini kit (Qiagen Sciences) with on column DNA digestion (DNA Free kit; Ambion) the three aliquots were combined onto a single column. RNA concentrations and purity were determined by measuring the absorbance at 260 nm, 280 nm and 230 nm using a NanoDrop ND-1000 spectrophotometer (NanoDrop Technologies). RNA quality was evaluated using the RNA 6000 NanoChip assay on a 2100 Bioanalyzer (Agilent Technologies). The 23 s:16 s rRNA ratio for all samples used exceeded 2.0.

### Microarray hybridization

Isolated total RNA (10 μg) was reverse-transcribed, fragmented using DNAseI and biotin end-labeled according to Affymetrix's Prokaryotic Target Labeling Protocol (GeneChip Expression Analysis Technical Manual; November, 2004). For each *Pseudomonas *genome array (#900339, Affymetrix), 4.5 μg of labeled fragmented cDNA was hybridized to the arrays at 50°C for 16 h with constant rotational mixing at 60 rpm. Washing and staining of the arrays was performed using the Affymetrix GeneChip Fluidics Station 450. Arrays were scanned using an Affymetrix GeneChip Scanner 7 G and GCOS software version 1.4. The MAS5 signal intensity for all the probes on the chip was determined.

### Comparison of rankings

Microarray data from studies of planktonic bacteria listed in Table 2 were used to interpret the data from our own microarrays. The available signal intensity data for all the probes on each microarray were downloaded from the NIH's gene expression omnibus (GEO) database and imported into Microsoft Excel along with our own microarray signal intensities. Our microarray data have been deposited in NCBI's Gene Expression Omnibus [92] and are accessible through GEO Series accession number GSE22164. For all of these data sets the probe intensities from each microarray were sorted from highest to lowest and the ranking for each of the loci of interest was taken as an average of the ranking from individual replicates.

Three of these data sets were repeatedly used as comparators; results of these particular comparators appear on most of the graphs in Figures 3, 5, and 6 and are the basis of the averaged comparator ranks reported in Table 3. These three data sets were the 20% oxygen condition of Alvarez-Ortega and Harwood [15]; the untreated control of Teitzel et al [20]; and the untreated control of Nalca et al. [18]. The first two were reported to be exponential phase cultures and the latter was described as an early stationary phase culture.

To compile the list of genes up-regulated in drip-flow biofilms, the average rank in the drip-flow biofilm data set was compared to the average rank in the three comparator data sets named above. The fold change in the rank between the biofilm and the planktonic comparators was calculated and the 100 genes with the highest fold change were tabulated.

### Statistics

Claims of statistically significant differences in transcriptome ranks are based on 109 individual two sample Welch *t*-tests (i.e. heterogeneous variances are modeled) on the ranks of each sample using a family-wise false discovery rate of 5% [93]. These analyses are similar to the non-parametric Friedman and Mack-Skillings rank tests used for the analysis of microarray data [94-97]. This approach is more conservative than the pooled *t*-test analysis of rank data advocated by Conover [98] since the Welch *t*-test models the obvious heteroscedastic variability between the ranks of the drip flow biofilm transcriptome and the ranks of the comparator transcriptomes.

## Authors' contributions

JF carried out the transcriptional profiling studies and helped to draft the manuscript. LR made measurements of biofilm antibiotic susceptibility and protein synthetic activity. BP assisted with microscopy. FR performed the oxygen microelectrode measurements. GE participated in the design of the study and formulation of hypotheses. AP performed the statistical analyses. AM performed the bioinformatic analysis that generated Figure 4. PS conceived the experimental and analytical approaches, supervised laboratory work and drafted the manuscript. All authors read and approved the final manuscript.

## Supplementary Material

Additional file 1 *P. aeruginosa *transcriptional profiling data sets used for comparison with colored symbol key. Click here for file
