# Supplementary material for: Physiology of Pseudomonas aeruginosa in biofilms as revealed by transcriptome analysis
Source: BMC Microbiol. 2010 Nov 17;10:294. doi: 10.1186/1471-2180-10-294 (PMC2998477; doi:10.1186/1471-2180-10-294)
Supplement: Additional file 1 — P. aeruginosa transcriptional profiling data sets used for comparison with colored symbol key. [file 1471-2180-10-294-S1.PDF]

Additional file 1. *P. aeruginosa* transcriptional profiling data sets used for comparison with colored symbol key.

GEO ID	Symbol Color	Medium	n	Reference
GSE6741	20% O_2 – light green<br>2% O_2 – gold<br>0.4% O_2 – red<br>0% O_2 + nitrate – dark green	minimal amino acids<br>37°C, sparged and stirred<br>exponential phase, OD ~<br>0.08	2	[15]
GSE2430	untreated control – pink	BHI, 37°C, shaken;<br>early stationary phase,<br>OD ~ 2.8	2	[18]
GSE4152	untreated control – yellow<br>Cu stressed – blue	MOPS buffered LB, 37°C,<br>early exponential phase,<br>OD ~ 0.2	2	[20]
GSE2885	OD ~ 0.2 – light gray<br>OD ~ 1.3 – white<br>OD ~ 2.1 (Fe limited) – purple	minimal glucose, 37°C,<br>sparged and stirred, three<br>points in batch culture	2	[22]
GSE5604	untreated control – light blue	minimal acetate, 20°C,<br>chemostat with dilution<br>rate 0.06 h^{-1}	2	[17]

GSE7704	● control – brown	minimal citrate, 37°C, shaken, OD ~ 0.6	3	[19]
GSE5443	● control – dark blue	LB, 37°C	2	[16]
GSE8408	● control – dark gray	minimal succinate and non-sulfur containing amino acids, 30°C, shaken, OD ~ 0.2	3	[21]
